# The effects of benralizumab on airway geometry and dynamics in severe eosinophilic asthma: a single-arm study design exploring a functional respiratory imaging approach

**DOI:** 10.1186/s12931-023-02415-4

**Published:** 2023-05-03

**Authors:** Eduardo Genofre, Donna Carstens, Wilfried DeBacker, Patrick Muchmore, Reynold A. Panettieri, Kirsty Rhodes, Vivian H. Shih, Frank Trudo

**Affiliations:** 1 BioPharmaceuticals Medical, US, AstraZeneca LP, 1800 Concord Pike, A1C, Wilmington, DE 19850 USA; 2 FLUIDDA, New York, NY USA; 3 Faculty of Medicine, University of Antwerp, Antwerp, Belgium; 4 Rutgers Institute for Translational Medicine and Science, Child Health Institute of New Jersey, Rutgers, The State University of New Jersey, New Brunswick, NJ USA; 5 BioPharmaceuticals Medical, AstraZeneca, Cambridge, UK; 6 BioPharmaceuticals Medical, AstraZeneca, Gaithersburg, MD USA

**Keywords:** Eosinophilic asthma, Functional respiratory imaging, Mucus plugging, Obstructive airway disease, Respiratory disease, T2 inflammation, Lung structure/function

## Abstract

**Background:**

Severe eosinophilic asthma (SEA) is characterised by elevated blood/sputum eosinophil counts and airway inflammation, which can lead to mucus plug-mediated airway obstruction, increased exacerbation frequency, declines in lung function, and death. Benralizumab targets the alpha-subunit of the interleukin-5 receptor found on eosinophils, leading to rapid and near complete eosinophil depletion. This is expected to result in reduced eosinophilic inflammation, reduced mucus plugging and improved airway patency and airflow distribution.

**Methods:**

BURAN is an interventional, single-arm, open-label, uncontrolled, prospective, multicentre study during which participants will receive three 30 mg subcutaneous doses of benralizumab at 4-week intervals. This study will use functional respiratory imaging (FRI), a novel, quantitative method of assessing patients’ lung structure and function based on detailed, three-dimensional models of the airways, with direct comparison of images taken at Weeks 0 and 13. Patients aged ≥ 18 years with established SEA who may be receiving oral corticosteroids and/or other asthma controller medications, who are inadequately controlled on inhaled corticosteroid-long-acting β_2_-agonist therapies and who have had ≥ 2 asthma exacerbations in the previous 12 months will be included. The objectives of BURAN are to describe changes in airway geometry and dynamics, measured by specific image-based airway volume and other FRI endpoints, following benralizumab therapy. Outcomes will be evaluated using descriptive statistics. Changes in FRI parameters, mucus plugging scores and central/peripheral ratio will be quantified as mean percent change from baseline (Week 0) to Week 13 (± 5 days) and statistical significance will be evaluated using paired t-tests. Relationships between FRI parameters/mucus plugging scores and conventional lung function measurements at baseline will be assessed with linear regression analyses for associations between outcomes, scatterplots to visualise the relationship, and correlation coefficients (Spearman’s rank and Pearson’s) to quantify the strength of these associations.

**Conclusions:**

The BURAN study will represent one of the first applications of FRI—a novel, non-invasive, highly sensitive method of assessing lung structure, function and health—in the field of biologic respiratory therapies. Findings from this study will increase understanding of cellular-level eosinophil depletion mechanisms and improvements in lung function and asthma control following benralizumab treatment.

*Trial registration* EudraCT: 2022-000152-11 and NCT05552508

## Background

Asthma is a chronic, heterogeneous, inflammatory condition of the airways, estimated to affect > 315 million people worldwide [1, 2]. Common symptoms include variable wheeze, shortness of breath, chest tightness and cough [2]. Eosinophilic asthma, characterised by elevated blood/sputum eosinophil counts [3–6], is the most common asthma phenotype, accounting for approximately 84% of all asthma cases and 50% of severe asthma cases [7–9]. The associated inflammatory cells, eosinophils, are terminally differentiated, bone marrow-derived granulocytes capable of secreting a panoply of mediators, growth factors and cytotoxic proteins critical to asthma-related increases in blood vessel permeability, leucocyte and plasma protein leakage into airways, mucus elasticity (via increased crosslinking) and mucus secretion [10–12]. Eosinophils are also the most common cell type found in the Charcot-Leyden crystals, which characterise the resulting thick, pathologic mucus that plugs the airways of patients with asthma [11, 13]. Unsurprisingly, blood/sputum eosinophil counts correlate with the severity of bronchoconstriction, mucus hypersecretion and thickening, airway inflammation and hyper-responsiveness, leading to potential airway remodelling [5, 12, 14, 15]. Sustained or severe elevations in eosinophil count can lead to mucus plug-mediated airway obstruction, air trapping, increased exacerbation frequency, declines in lung function and even death [10, 13, 15–17].

As elevated blood eosinophil counts and eosinophil infiltration into the airways are closely linked with asthma severity [2, 5, 6, 10, 18–20], the depletion of eosinophils is a goal of eosinophilic asthma management. It is thought that this will lead to a reduction in inflammation and mucus plugging, and therefore an improvement in airway patency and airflow distribution. To this end, a number of eosinophil-targeting biologic therapies, including benralizumab, have been developed [5, 20–23].

Benralizumab is a subcutaneously administered, humanised monoclonal antibody approved for use in the USA, Europe and other nations for the treatment of patients with severe eosinophilic asthma (SEA) [4, 5, 19, 20, 24]. It directly and specifically targets the alpha-subunit of the interleukin-5 receptor (IL-5R) found on eosinophils, leading to rapid and near complete eosinophil depletion by natural-killer cell-mediated apoptosis [5, 25, 26]. Other biologics (e.g. mepolizumab, reslizumab), by comparison, target the IL-5R ligand (IL-5), and result in reductions in, rather than depletion of, eosinophils [5, 20, 22, 23]. Furthermore, because benralizumab is delivered subcutaneously, it has the potential to quickly reach areas of the lungs inaccessible to inhaled therapies as a result of inflammation or mucus plugs [16, 27, 28].

The actions of benralizumab at cellular and clinical levels are fairly well understood [4, 5, 18, 19] and multiple phase 3 clinical studies (SIROCCO, CALIMA, ZONDA, BORA, MELTEMI) have demonstrated its ability to rapidly, efficiently, and safely achieve long-term eosinophil depletion to near undetectable levels [19, 20, 24, 29, 30].

Understanding the functional changes in airway geometry and dynamics has proven to be a challenge, with few sensitive, non-invasive methods of accurately determining the location, extent, progression and treatment responses of lung pathology [27, 28, 31]. Common spirometry endpoints in clinical trials, such as improvement in forced expiratory volume in 1 s (FEV_1_), do not necessarily correlate with improvements in patient-reported outcomes (PROs) [27, 28]. Current standard methods of imaging the lungs are limited by poor resolution and difficulty of interpretation (X-ray), concerns surrounding radiation exposure (nuclear medicine-based methods such as positron emission tomography), or the need for an inhaled contrast medium (used in some magnetic resonance imaging techniques) [16, 31].

The novel technique of functional respiratory imaging (FRI), by comparison, enables a comprehensive assessment of pulmonary function by providing detailed, accurate, comparable anatomic/structural images and dynamic airflow parameters from multiple time points (i.e. before and during benralizumab therapy) [31–35]. This will allow us to investigate the as-yet unknown short-term effects of benralizumab-mediated sustained airway eosinophil depletion on airway dynamics (including inflammation and mucus plugging). Furthermore, FRI provides an opportunity to assess the extent to which systemic therapies such as benralizumab may produce indirect improvements in airway patency and airflow dynamics by increasing the access of inhaled therapy to affected tissues [21].

## Functional respiratory imaging (FRI)

FRI is a novel, non-invasive, quantitative method of assessing lung structure and function based on detailed, three-dimensional (3D) models of the airways of individual patients [21, 31], derived from high-resolution computed tomography (HRCT) and cryo-fluorescence tomography (CFT) images [31]. FRI uses imaging equipment available at many hospitals, is not reliant on inhaled media, and allows images to be obtained at any point in the respiratory cycle [15, 32]. Furthermore, segmentation algorithms can extract data from regions of interest (lobes, airways, blood vessels), permitting investigators to isolate the early stage effects of pathologies and therapies on specific systems and areas of the lungs [31]. Multiple studies have validated FRI endpoints in terms of their correlations with changes in PROs and pulmonary function tests (PFTs) across a range of aetiologically distinct conditions [31, 34–38], suggesting that this technology could help improve understanding of SEA pathogenesis, disease progression and treatment efficacy [38, 39].

It is also possible to simulate airflow and pressure, particle density and distribution by applying computational fluid dynamics (CFD) calculations (based on Navier–Stokes equations) to the airway models produced by FRI. From these, functional parameters such as airway resistance, airflow distribution and inhaled particle deposition can be extracted, potentially enabling the detection of improvements in inhaled therapy deposition resulting from injected (i.e. benralizumab) or oral (i.e. prednisolone) therapies [21, 27, 28, 31, 33].

FRI endpoints used in clinical trials include lung and lobular volume (iVlung and iVlobe), airway volume (iVaw), internal airflow distribution (IAD), airway resistance (iRaw), blood vessel volume (BVX), mucus plugging score and air trapping score.

### iVlung and iVlobe

iVlung and iVlobe are summations of voxels (volumetric [3D] pixels) representing the air in the lungs, and provide total lung volume and the volume of each lobe (using the fissure planes on CT images to segment lobes), respectively [31, 36]. While iVlung offers an approximation of lung capacity, iVlobe provides insights into the extent/location of respiratory illnesses and data useful for establishing air flow distribution [36, 38]. However, the utility of iVlung and iVlobe is limited due to the lack of research examining their correlations with parameters such as sex, height, and ethnicity [31, 40, 41].

### iVaw

Airway volume can be assessed at the individual airway level, allowing for quantification of volumetric changes resulting from inhalation and exhalation, and identification and appraisal of airway obstructions (e.g. mucus plugs), expansions (i.e. bronchiectasis) and constrictions (e.g. muscular hypertrophy, fibrosis) [31, 36].

To further simplify comparisons between patients, a ‘specific’ airway volume (siVaw) parameter is used—the ratio of airway to lung volume—which not only corrects for inter-patient differences in lung volume, but also reflects the positive relationship between iVaw and iVlobe [31, 32].

### IAD

By assessing changes in iVlobe between total lung capacity (TLC) and functional residual capacity (FRC), patient-specific airflow distribution can be established. This not only contributes to the identification of areas of narrowed or obstructed airways, but is also one of the parameters necessary to calculate deposition of aerosols in the lungs [31].

### iRaw

iRaw accounts for patient-specific airflow distribution (since air will tend to flow from areas of higher pressure, i.e. resistance, to areas of lower pressure), and is calculated (via CFD) as the total pressure drop within an airway, divided by the flow rate through that airway [31, 36]. Increases in airway resistance can be indicative of inflammation or fibrosis [38], whereas decreases can be suggestive of a good response to bronchodilatory agents [34, 36]. While iRaw accounts for 80% of airway resistance, the remaining 20% resulting from small airway resistance can only be approximated from iVlobe [31, 32, 36].

### iVaww

iVaww (considered the sum of all imaged tissue that encompasses airway walls) provides the volume of the airway walls, and therefore can indicate the severity and extent of pathologic concerns such as inflammation or fibrosis. iVaww also has a bearing on iRaw, since thickened airway walls result in greater airway resistance [31].

### BVX

BVX refers to the cross-sectional area of vasculature used to calculate blood vessel volumes; specific values include BV5, BV5_10 and BV10, which relate to blood volume in vessels of ≤ 5 mm^2^, 5–10 mm^2^ and > 10 mm^2^ diameter, respectively [42]. The areas and volumes themselves are obtained via 3D reconstruction of segmented blood vessels at TLC [31]. These measures are essentially a surrogate for perfusion, and therefore the capacity of injected therapies to access lung tissue [27]. Changes in vessel calibre and the corresponding redistribution of pulmonary blood volume may also provide an indication of treatment effectiveness, since localised pulmonary vasoconstriction is associated with regions of alveolar hypoxia secondary to asthmatic bronchoconstriction [43].

### Mucus plugging score

Mucus plugging can be assessed in segmented airways by manually searching for areas of obstructed airway bracketed by sections of clear airway, thus allowing for the identification of multiple, sequential blockages [10]. The segments of each lobe are scored as 0 or 1 according to the presence or absence of mucus plugs. Per-segment scores for each lobe are then summed to yield a total mucus plugging score for both lungs (ranging from 0 to 20), which could be used as a quantitative measure of disease severity or treatment efficacy in conditions where mucus plugging is a concern (i.e. asthma, cystic fibrosis [10, 15, 44, 45]). Since the score may be impacted by airway obstruction from causes unrelated to mucus plugging, overestimation may occur, which is a potential limitation of this measure [44].

### Air trapping score

Abnormal retention of air can be visualised and assessed across the volume of the lungs as all intrapulmonary voxels between − 1024 and − 850 Hounsfield Units at FRC [31, 32], and may account for increases in FRC and reductions in inspiratory capacity associated with airway obstruction in chronic obstructive pulmonary disease (COPD) [38] and asthma [46]. Air trapping score could also provide an approximation of the level of mucus plugging and inflammation in an asthmatic lung, and validate changes in mucus plugging score. The score itself is based on a five-point scale: 0: no air trapping; 1: 1% to 25%; 2: 26% to 50%; 3: 51% to 75%; and 4: 76% to 100% of the cross-sectional areas of the affected lung. The total air trapping score is the sum of the scores measured at different cross-sections.

### Aerosol deposition

The effects of benralizumab therapy on inhaled aerosol deposition, defined as the distribution, level of airway infiltration, and regional concentration of inhaled aerosols—the effective lung dose of inhaled medication [27, 32, 47, 48]—can also be examined using FRI. Deposition is calculated based on CFD, IAD, patient-specific airway geometries and particle size data from an idealised dose of inhaled therapy. Simulated particles are deposited into simulated airflow and any calculated particle with a trajectory intersecting the airway wall is considered trapped at that location [31–33, 48]. FRI has a high level of agreement with isotope-based methods of studying aerosol deposition (gamma scintigraphy and single-photon emission CT) [33], but accurate readings require detailed knowledge of particle distribution for individual inhalers and good inhalation technique [48].

Relative drug deposition in the intrathoracic versus peripheral airways will be assessed using the central/peripheral (C/P) drug deposition ratio. Central airways are defined as the trachea and all airways with diameter > 1–2 mm and reaching out to the 7–10th airway generation, while the peripheral airways are defined as airways with a diameter < 1–2 mm and > 10 generations of branching.

In summary, FRI has the potential to permit direct, non-invasive observation of respiratory geometries and dynamics, and therefore the effects of various factors upon them, via the direct comparison of images taken at different time points [27, 31, 38]. Given this, we hypothesise that the FRI-enabled quantification of airway geometry and dynamics will reveal how lung physiology and function in the asthmatic lung correlate with PFTs and PROs. We also hypothesise that FRI can reveal relationships between changes in the values of imaged parameters (airway geometries and dynamics) and improvements seen in PFTs and PROs from the early stages of benralizumab therapy.

The BURAN study aims to use the novel FRI imaging technology to explore the early, short-term impact of benralizumab treatment on lung physiologic and functional parameters. In addition, it aims to explore correlations between these imaged parameters and benralizumab-induced changes in PROs and PFTs.

## Methods and analysis

### Patients

The BURAN study population will comprise male and female patients aged 18–70 years with established SEA (as defined by European Respiratory Society/American Thoracic Society [ERS/ATS] clinical guidelines [49]), inadequately controlled by inhaled corticosteroids-long-acting β_2_-agonists (ICS-LABA) with or without oral corticosteroids (OCS) and/or other asthma controller medications.

**Fig. 1:**
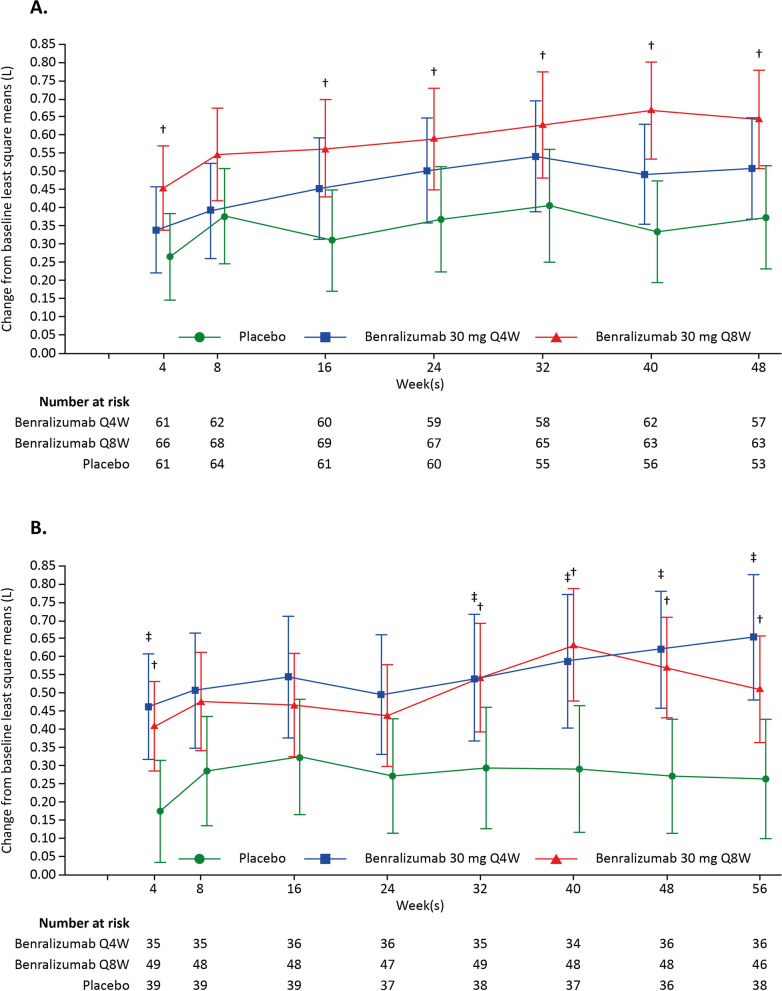
Change from baseline FEV_1_ in the SIROCCO (**A**) and CALIMA (**B**) trials * [20, 24]. *All patients had BEC ≥ 300 cells/µL, documented high-dose ICS-LABA ≥ 12 months, and pre-BD FVC < 65% predicted. ^†^p < 0.05 for benralizumab 30 mg Q8W vs placebo. ^‡^p < 0.05 for benralizumab 30 mg Q4W vs placebo. Error bars represent 95% confidence intervals. *BD* bronchodilator, *BEC* blood eosinophil count, *FEV*_*1*_ forced expiratory volume in 1 s, *FVC* forced vital capacity, *ICS* inhaled corticosteroid, *LABA* long-acting β_2_-agonist, *Q4W* every 4 weeks, *Q8W* every 8 weeks

Data from the SIROCCO and CALIMA studies indicate that patient populations with more severe SEA (blood eosinophil count [BEC] ≥ 300 cells/µL, documented high-dose ICS-LABA ≥ 12 months, pre-bronchodilator [BD] forced vital capacity [FVC] < 65% predicted) have an enhanced response to benralizumab therapy with respect to FEV_1_ (Fig. 1 [20, 24]). Therefore, the BURAN study population, while similar to those in SIROCCO and CALIMA, will be enriched by including patients with more compromised lung function. This will provide a clearer picture of benralizumab-related changes in airway geometries and dynamics and enable use of a smaller patient population to observe significant results.

Eligible patients must have had ≥ 2 asthma exacerbations in the last 12 months, where an exacerbation is defined as a worsening of asthma that leads to any of the following:

1. Use or increased use of systemic corticosteroids (SCS) for ≥ 3 days (for these purposes, a single depot-injectable dose of corticosteroids will be counted as equivalent to a 3-day course of SCS);
2. Evaluation and treatment at an emergency department (ED) or urgent care centre for < 24 h, leading to SCS treatment per point 1;
3. Admission to an inpatient facility and/or evaluation and treatment in a healthcare facility for ≥ 24 h.

All patients must also demonstrate:

- Pre-BD FVC < 65% predicted and FEV_1_ < 80% predicted;
- Asthma Control Questionnaire-6 (ACQ-6) score ≥ 1.5;
- Post-BD asthma reversibility (≥ 12% and ≥ 200 mL in FEV_1_ and/or FVC over baseline following short-acting β_2_-agonist [SABA] inhalation);
- BEC either ≥ 150 cells/µL (if OCS dependent) or ≥ 300 cells/µL (if not OCS dependent);
- Evidence of a stable asthma treatment regime (excluding rescue medication use), including documented high-dose ICS-LABA for ≥ 3 months prior to Visit 0 (V0; Screening visit) and stable OCS dosage for ≥ 4 weeks prior to V0;
- Ability to tolerate withdrawal of asthma medication for at least 24 h prior to Visits 0, 1, and 4 (V0, V1, and V4; Screening visit, Week 0, and Week 13), without worsening of symptoms.

Exclusion criteria include:

- Diagnosis or suspicion of clinically relevant respiratory or non-respiratory conditions or anomalies (including COVID-19 and malignancy);
- Historic or current alcohol or other substance abuse considered likely to affect compliance with study measures;
- Use of any biologic asthma therapy ≤ 4 months/5 half-lives prior to V0 or any live-attenuated vaccine ≤ 30 days prior to V0;
- Known hypersensitivity/anaphylaxis to any vaccine or biologic therapy (including benralizumab).

For detailed inclusion and exclusion criteria (based on SIROCCO and CALIMA analyses) see Table 1.

**Table 1:** Inclusion and exclusion criteria

**Inclusion criteria**
• Adult patients (aged 18–70 years) • Diagnosed with asthma according to ATS/ERS guidelines [49] • Free from concomitant treatments (including oral/ophthalmic β-blockers and/or immunosuppressants) or comorbid conditions likely to interfere with study procedure or increase participation-related risks • Documented reversibility (≥ 12% and ≥ 200 mL in FEV_1_ and/or FVC over baseline) following SABA inhaler use [52] • ≥ 2 exacerbations in the 12 months prior to V0 (the last one ≤ 6 weeks prior to V0) • Able to perform acceptable and repeatable spirometry according to ATS/ERS 2019 [55] or protocol-defined criteria • Able to understand and comply with PRO questionnaires, protocol restrictions and schedule • FVC < 65% predicted, FEV_1_ < 80% predicted and ACQ-6 score ≥ 1.5 at V0 • Documented high-dose ICS-LABA use ≥ 3 months before V0, with or without OCS and additional asthma controllers • Stable asthma treatment regime (excluding rescue medication use) ≥ 3 months prior to V0 • Tolerant of asthma medication withdrawal for at least 24 h prior to V0, 1 and 4 • Documented peripheral BEC ≥ 300 cells/μL or ≥ 150 cells/μL (if OCS dependent) at V0; in study sites in Belgium, all patients must have BEC ≥ 300 cells/μL, irrespective of OCS dependency • Fully vaccinated against COVID-19
**Exclusion criteria**
• Exacerbation or acute airway infection ≤ 6 weeks prior to V0 • Positive COVID-19 test based on prevailing local guidelines for COVID-19 testing, COVID-19 infection ≤ 6 weeks before screening or severe COVID-19 at any time • Current malignancy or historic malignancy in remission ≤ 5 years prior to V0 (excepting localised basal/squamous cell carcinoma OR in situ cervical carcinoma in remission ≥ 12 months prior to V0) • Diagnosis of any relevant non-asthma pulmonary disease or any condition associated with peripheral eosinophilia • Diagnosis of any other disease or abnormality likely to affect study analyses or increase participation-related risks • Current or historic alcohol or substance abuse judged likely to affect study analyses or compliance • Scheduled inpatient surgery or other admission to hospital • Use of any biologic asthma therapy ≤ 4 months/5 half-lives prior to V0 • Use of any live-attenuated vaccine ≤ 30 days prior to V0 • History of lung volume reduction surgery, lung resection or thermal bronchoplasty at any time before V0 • Participation in another clinical study ≤ 4 months (biologics), ≤ 30 days (non-biologic), or ≤ 5 half-lives prior to V0 • Known hypersensitivity/anaphylaxis to any vaccine or biologic therapy (including benralizumab) • Involvement in the planning or conduct of this study or any immediate relation to study personnel

*ACQ-6* Asthma Control Questionnaire-6, *ATS* American Thoracic Society, *BD* bronchodilator, *BEC* blood eosinophil count, *COVID-19* coronavirus disease 2019, *ERS* European Respiratory Society, *FEV*_*1*_ forced expiratory volume in 1 s, *FVC* forced vital capacity, *ICS* inhaled corticosteroid, *LABA* long-acting β_2_-agonist, *OCS* oral corticosteroid, *PRO* patient-reported outcome, *V* visit

### Study design

**Fig. 2:**
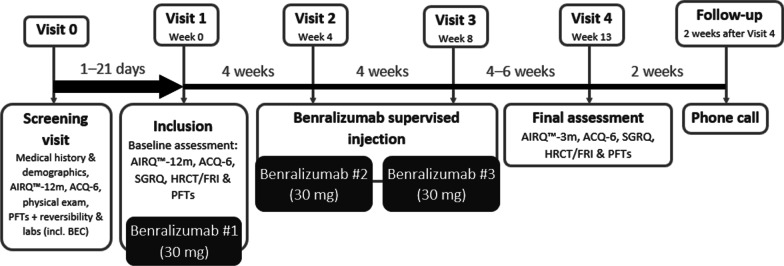
BURAN study design. ACQ-6, Asthma Control Questionnaire-6; AIRQ™-3 m, Asthma Impairment and Risk Questionnaire (3-month intervals); AIRQ™-12 m, Asthma Impairment and Risk Questionnaire (12-month intervals); BEC, blood eosinophil count; FRI, functional respiratory imaging; HRCT, high-resolution computed tomography; PFT, pulmonary function test; SGRQ, St George’s Respiratory Questionnaire

BURAN is an interventional, single-arm, open-label, uncontrolled, prospective, multicentre clinical trial. All patients approved to participate in the study will receive three 30 mg subcutaneous doses of benralizumab, per US Food and Drug Administration-/European Medicines Agency-approved dosing schedules, delivered as a 1 mL solution in a single-use pre-filled glass syringe (Fig. 2). To establish baseline measures of lung function, maintenance and rescue medications will be withdrawn for up to 24 h ahead of V0, V1 and V4 (see Table 2).

**Table 2:** Withdrawal periods for maintenance and rescue therapies prior to V0, V1, and V4

Pharmaceutical class	Withdrawal period
SABAs	≥ 6 h
SAMAs	≥ 12 h
SAMA/SABA combinations	≥ 12 h
SMART/MART	≥ 12 h 1. SMART/MART reliever therapy and the use of (budesonide/formoterol) as a rescue therapy are permitted at investigator discretion 2. Total ICS dose resulting from permitted SMART/MART use must be recorded
LABA-containing therapies	1. Once daily: ≥ 24 h 2. Twice daily: ≥ 12 h
LAMA-containing therapies	1. Once daily: ≥ 24 h 2. Twice daily: ≥ 12 h
LTRAs	≥ 24 h
Theophyllines	1. Once daily: ≥ 24 h 2. Twice daily: ≥ 12 h

*ICS* inhaled corticosteroid, *LABA* long-acting β_2_-agonist, *LAMA* long-acting muscarinic antagonist, *LTRA* leukotriene receptor antagonist, *MART* maintenance and reliever therapy, *SABA* short-acting β_2_-agonist, *SAMA* short-acting muscarinic antagonist, *SMART* single maintenance and reliever therapy, *V* visit

### Study timeline

Planned duration of treatment in BURAN was informed by the SIROCCO and CALIMA studies [20, 24], which showed that response to benralizumab therapy is both faster in higher severity populations (as early as 4 weeks) and sustained for ≥ 12 weeks with maintenance dosing every 8 weeks. Participants will therefore be enrolled in the study for 15–23 weeks, inclusive of study follow-up, during which time procedures and assessments will be performed, as outlined in Fig. 2.

### Study endpoints

All comparisons between outcome measures will be calculated within-patient. Baseline will be considered to be Week 0 and the post-treatment period, Week 13 (± 5 days; see Fig. 2).

The primary objective of the BURAN study is to describe changes in airway dynamics following benralizumab therapy, as measured by siVaw. Therefore, the primary endpoint will be the percent change from baseline to Week 13 in siVaw at TLC.

The main secondary objective is to describe changes in airway dynamics, following benralizumab therapy, as measured by other FRI endpoints. Therefore, secondary endpoints will be the percent changes from baseline to Week 13 in the following imaging endpoints: iVlung, iVlobe, iVaww, iRaw, IAD, BVX, air trapping score, mucus plugging score, ventilation mapping and ventilation/perfusion mapping (all measured at TLC).

The additional secondary objective is to describe the relationship between airway dynamics and conventional measurements of respiratory function (PFTs), both at baseline and according to treatment response. This will be carried out using measures of correlation between imaging endpoints (FRI endpoints [siVaw, iVlung, iVlobe, iVaww, air trapping, iRaw, BVX, IAD, ventilation mapping and ventilation/perfusion mapping] and mucus plugging score) and pre-BD FEV_1_ and FVC, both at baseline and according to treatment response (changes from baseline to Week 13).

Exploratory objectives are to evaluate correlations between imaging endpoints (FRI endpoints and mucus plug score) and PRO questionnaires (ACQ-6, St George’s Respiratory Questionnaire [SGRQ, total and component scores], Asthma Impairment and Risk Questionnaire [AIRQ]™-3 m and AIRQ™-12 m [the 3- and 12- month variants of AIRQ™]) at baseline and according to treatment response (changes from baseline to Week 13).

Safety endpoints in BURAN will include the frequency, intensity, severity, and outcome (including mortality and discontinuation) of any adverse event (AE), and its relationship (as judged by investigators) to the study procedure. Heart rate, and systolic and diastolic blood pressure will also be monitored.

### Spirometry and PROs

Spirometry will be performed according to 2005 ERS/ATS standard guidelines [52]. Pre-BD spirometry will be conducted at V0, V1 and V4 (see Fig. 2) after imaging procedures, to reduce the potential effects of spirometry on FRI parameters. Post-BD spirometry will only be performed at V0 and only in patients who do not have previously documented post-BD reversibility. The highest technically acceptable pre- and/or post-BD FEV_1_ and FVC will be recorded.

All PRO questionnaires will be completed by all participants at study centres using an electronic device at V0, V1 and V4, prior to the initiation of any other procedure. ACQ-6 will be collected across all three of these visits, SGRQ at V1 and V4, AIRQ™-12 m at V1 and AIRQ™-3 m at V4.

### Imaging methodology

FRI parameters will be obtained using low-dose HRCT (≥ 64-slice, or capable of acquiring 64 slices of thickness < 0.625 mm) thorax scans of study participants and a low radiation scanning protocol. All participants in the study will undergo two HRCT scans at V1 and V4 (one at TLC and one at FRC) with respiratory gating. Image analysis will be performed using the Mimics medical image processing software package (Materialise, Leuven, Belgium) by trained FRI analysts. Output will include patient-specific, segmented, 3D computer models of the lung lobes, airway lumen and wall, and vascular tree, from which structural and functional parameters can be extracted. Details of parameter calculation methodology can be found above.

### Discontinuation and adverse event reporting

Discontinuation of treatment will occur in the event of any of the following:

- Patient request;
- Any AE judged to threaten patient safety;
- Any asthma-related event resulting in hospitalisation for > 24 h or intubation, or requiring new OCS use after Week 11;
- Development of anaphylaxis, severe helminth infection, or new or recurrent malignancy, except non-melanoma skin cancers;
- Severe failure to comply with study requirements;
- Any positive pregnancy or COVID-19 test.

Severe adverse events (SAEs) will be recorded from receipt of informed consent, and AEs will be recorded from V1. All AEs will be followed up for as long as medically indicated. Causal relationships between AEs and the study procedure will be established in all cases. All SAEs, especially those with suspected causal links to benralizumab, will be reported to the study sponsor within 24 h.

### Statistical considerations

The study populations will be as follows:

- *Study population (evaluable)*: All patients who received three doses of benralizumab, and underwent baseline and treatment study evaluation (PROs, CT scans and spirometry).
- *Primary analysis population*: The proportion of the study population who did not experience acute asthma exacerbations or lower respiratory tract infections during the study period.
- *Safety analysis set*: All patients who received at least one dose of benralizumab.
- *Baseline endpoints analysis set*: Patients who underwent baseline measurements and who had received ≥ 1 dose of benralizumab.

All outcomes will be evaluated using descriptive statistics. Continuous variables will be summarised as means, standard deviations, 95% confidence intervals, or medians and inter-quartile ranges. Categorical variables will be reported as numbers and percentages among complete patient data sets. Changes in FRI parameters, mucus plugging scores and central/peripheral ratio will be quantified as the mean percent change from baseline (Week 0) to Week 13 (± 5 days). Paired t-tests will then be used to determine if there are any statistically significant differences between the means of each outcome measure at baseline and Week 13, where the level of statistical significance will be set at p = 0.05. Scatterplots will be plotted to display FRI parameters/mucus plugging score versus conventional lung function measurements at baseline (Week 0), providing an early indication of the relationship between these endpoints. Correlation coefficients (Spearman’s rank and Pearson’s, as appropriate) will quantify the strength of any associations. A linear regression model will be fitted to estimate the mean increase in FRI parameters/mucus plugging scores per unit increase in conventional lung function measurements, with 95% confidence intervals.

The relationship between changes from baseline to Week 13 in FRI parameters/mucus plugging scores and conventional measures of lung health/function will be assessed, again, using scatterplots to visualise the relationships and correlation coefficients (Spearman’s rank and Pearson’s, as appropriate) to quantify the strength of the associations. Linear regression analyses will be applied to compare outcomes at Week 13 versus Week 0, with and without adjustment for pre-BD FEV_1_ and FVC. Results of the unadjusted and adjusted models will be compared to indicate whether changes from baseline to Week 13 in the FRI parameters/mucus plugging scores can be explained by changes from baseline in pre-BD FEV_1_ and pre-BD FVC.

**Fig. 3:**
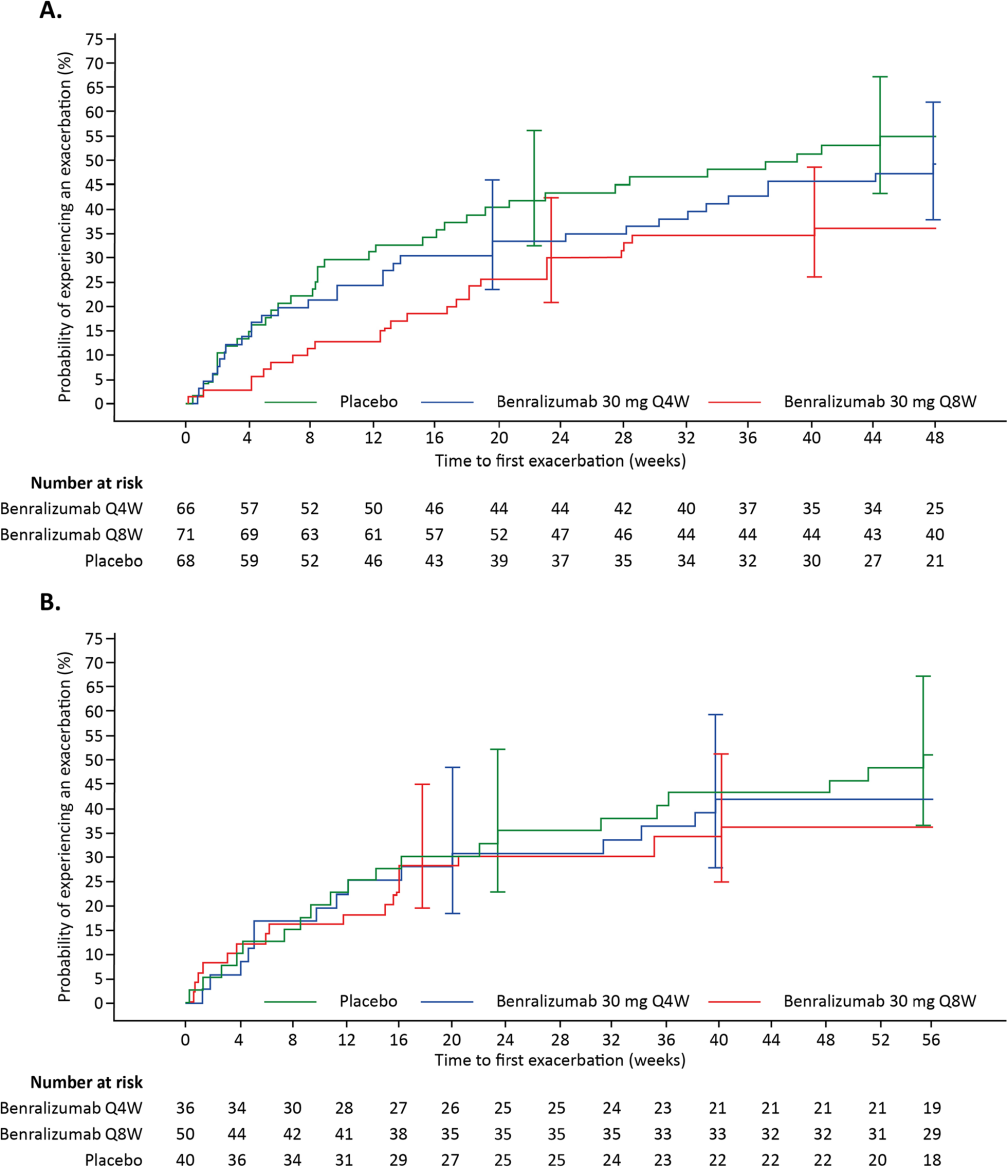
Time to first exacerbation in the SIROCCO (**A**) and CALIMA (**B**) trials * [20, 24]. *All patients had BEC ≥ 300 cells/µL, documented high-dose ICS-LABA ≥ 12 months, and pre-BD FVC < 65% predicted. BD, bronchodilator; BEC, blood eosinophil count; FVC, forced vital capacity; ICS, inhaled corticosteroid; LABA, long-acting β_2_-agonist; Q4W, every 4 weeks; Q8W, every 8 weeks

A minimum of 138 patients will be enrolled to achieve a minimum of 126 evaluable participants (107 participants for the primary analysis), assuming a 10% dropout rate and a 15% probability of experiencing an exacerbation during the study (based on results from equivalent CALIMA and SIROCCO populations (Fig. 3; [20, 25]).

## Discussion

We present the design of the BURAN study, which will use FRI—a novel, non-invasive, highly sensitive method of assessing geometric and functional respiratory parameters—to examine the effects of benralizumab on lung health. This new approach will allow direct, high-resolution observation of the health and function of the living asthmatic lung, including levels of inflammation, location and prevalence of mucus plugs, distribution of airflow (therefore access of inhaled therapies to the target organ) and how the above change in response to factors ranging from physiology, to exacerbation, to therapy [21, 31–34, 36, 47]. This represents a significant improvement in the volume, diversity, precision, and accuracy of data compared with that offered by current best practice techniques (i.e. spirometry outcomes and PROs) for quantifying lung health, which tend to provide indirect and/or subjective information [31]. For example, van Geffen et al. were able to identify regionalised changes in ventilation and resistance in the lungs of patients experiencing acute COPD exacerbations, information which they hypothesised could be used to identify exacerbation phenotypes and therefore guide therapy [38]. Examples of another sort can be found in a number of studies demonstrating the ability of FRI to quickly, accurately and precisely assess the deposition of inhaled treatment and its effects in the lung [32–34, 36, 47]. Furthermore, a number of authors have reported the successful use of FRI to predict treatment outcomes in respiratory conditions as diverse as sleep apnoea and lung cancer [31]. FRI can therefore be considered a potent tool for the identification and treatment of respiratory diseases, potentially allowing individualisation and rapid adjustment of therapies.

We would like to address several details of the study design. Firstly, the study will be conducted as single-arm (uncontrolled). Whilst this could be considered concerning, the efficacy and safety of benralizumab are well established [5, 18, 20, 29, 30] and the intention of this study is to establish the airway level mechanisms underlying this confirmed efficacy. Furthermore, FRI-based endpoints are not influenced by effort-related placebo effects [34, 47], and the recruitment of a placebo arm would expose more patients than strictly necessary to imaging-associated radiation and worsening asthma symptoms.

Secondly, changes in siVaw were chosen as our primary outcome measure as they reflect per-patient changes in the lung-wide level of inflammation [31], hence functional respiratory changes, and therefore changes in health and quality of life. Current state-of-the-art CT scanners do not, however, provide resolution of airways < 1–2 mm in diameter [31, 32, 36], which represent many of those affected by obstructive respiratory diseases [53, 54]. Therefore, we have included parameters such as iVlobe and IAD, which are not CT-resolution dependent, in order to produce the widest possible picture of benralizumab’s respiratory effects [31]. Additionally, while PROs are primarily included in the BURAN study with the intention of understanding the relationships of patient symptoms and health-related quality of life to airway dynamics, the PROs in this study often have similar questions. The repetitive nature of these questions may lead to patient burden and potential discrepancies in patient responses across the tools; however, all PROs are quick to complete and provide pertinent sources of information to further validate the AIRQ™ for use in future asthma clinical studies [50, 51].

Thirdly, patients enrolled in this study will be required to have a peripheral BEC of ≥ 300 cells/μL (≥ 150 cells/μL if OCS dependent), an inclusion criterion set to ensure that all patients recruited display an eosinophilic asthma phenotype. Once the nature of benralizumab-mediated changes in respiratory geometry, structure and function have been established at higher BECs, variations related to lower BECs can be investigated.

Lastly, we acknowledge that this study concerns only the short-term effects of benralizumab, in line with previous findings demonstrating its fast onset of effect [5, 18, 20, 24]; future studies may use FRI to scrutinise long-term physiological effects.

## Conclusions

We aim to improve understanding of the eosinophil-depleting effects of benralizumab on airway structure and dynamics, including the level of mucus plugging and deposition of inhaled medications. It is also anticipated that the BURAN study will provide insights into the relationship between changes in PROs, PFTs, and airway dynamics and structure. Our results will help further characterise physiologic changes resulting from eosinophil depletion with benralizumab and better delineate the impact of these changes on PROs and PFTs. Whilst the BURAN study implements FRI primarily to examine the effects of benralizumab at the airway level, it is likely that it will be widely adopted as a research and healthcare tool in the future.

## Data Availability

The datasets used and analysed during the current study may be obtained in accordance with AstraZeneca’s data sharing policy, described at https://astrazenecagrouptrials.pharmacm.com/ST/Submission/Disclosure.

## Abbreviations

3D: Three-dimensional
ACQ-6: Asthma Control Questionnaire-6
AE: Adverse event
AIRQ™-3 m: Asthma Impairment and Risk Questionnaire (3-month interval)
AIRQ™-12 m: Asthma Impairment and Risk Questionnaire (12-month interval)
ATS: American Thoracic Society
BD: Bronchodilator
BEC: Blood eosinophil count
BVX: Blood vessel volume
C/P: Central/peripheral deposition ratio
CFT: Cryo-fluorescence tomography
CT: Computed tomography
ER: Emergency room
ERS: European Respiratory Society
FEV_1_: Forced expiratory volume in 1 s
FRC: Functional residual capacity
FRI: Functional respiratory imaging
FVC: Forced vital capacity
HRCT: High-resolution computed tomography
HU: Hounsfield units
IAD: Internal airflow distribution
ICS: Inhaled corticosteroid
iRaw: Imaged airway resistance
iVaw: Imaged airway volume
iVaww: Imaged airway wall volume
iVlobe: Imaged lobe volume
iVlung: Imaged lung volume
LABA: Long-acting β_2_-agonist
LAMA: Long-acting muscarinic antagonist
LTRA: Leukotriene receptor antagonist
MART: Maintenance and reliever therapy
OCS: Oral corticosteroid
PFT: Pulmonary function test
PRO: Patient-reported outcome
Q4W/q.4: Every 4 weeks
Q8W/q.8: Every 8 weeks
SABA: Short-acting β_2_-agonist
SAE: Severe adverse event
SAMA: Short-acting muscarinic antagonist
SEA: Severe eosinophilic asthma
SGRQ: St George’s Respiratory Questionnaire
siVaw: Specific imaged airway volume
SMART: Single maintenance and reliever therapy
TLC: Total lung capacity
V: Visit
